# A novel assay of antimycobacterial activity and phagocytosis by human neutrophils

**DOI:** 10.1016/j.tube.2012.11.014

**Published:** 2013-03

**Authors:** David M. Lowe, Nonzwakazi Bangani, Meera R. Mehta, Dirk M. Lang, Adriano G. Rossi, Katalin A. Wilkinson, Robert J. Wilkinson, Adrian R. Martineau

**Affiliations:** a Department of Medicine, Imperial College London, W2 1PG, UK; b Clinical Infectious Disease Research Initiative, Institute of Infectious Diseases and Molecular Medicine, University of Cape Town, Observatory 7925, South Africa; c Department of Human Biology, Faculty of Health Sciences, University of Cape Town, Observatory 7925, South Africa; d Medical Research Council Centre for Inflammation Research, The Queen's Medical Research Institute, University of Edinburgh, Edinburgh EH16 4TJ, Scotland, UK; e Division of Mycobacterial Research, MRC National Institute for Medical Research, The Ridgeway, Mill Hill, London NW7 1AA, UK; f Queen Mary University of London, Blizard Institute, London E1 2AB, UK

**Keywords:** Tuberculosis, Granulocytes, Bio-luminescence, Flow cytometry

## Abstract

Despite abundant evidence that neutrophils arrive early at sites of mycobacterial disease and phagocytose organisms, techniques to assay phagocytosis or killing of mycobacteria by these cells are lacking. Existing assays for measuring the antimycobacterial activity of human leukocytes require cell lysis which introduces new bioactive substances and may be incomplete. They are also time-consuming and carry multiple risks of inaccuracy due to serial dilution and organism clumping. Flow cytometric techniques for measuring phagocytosis of mycobacteria by human cells have failed to adequately address the effects of organism clumping, quenching agents and culture conditions on readouts.

Here we present a novel in-tube bioluminescence-based assay of antimycobacterial activity by human neutrophils. The assay yields intuitive results, with improving restriction of mycobacterial bioluminescence as the ratio of cells to organisms increases. We show that lysis of human cells is not required to measure luminescence accurately.

We also present a phagocytosis assay in which we have minimised the impact of mycobacterial clumping, investigated the effect of various opsonisation techniques and established the correct usage of trypan blue to identify surface-bound organisms without counting dead cells. The same multiplicity of infection and serum conditions are optimal to demonstrate both internalisation and restriction of mycobacterial growth.

## 1 Introduction

Tuberculosis is a major threat to humanity, but the host response to *Mycobacterium tuberculosis* remains incompletely understood. The role of neutrophils is particularly controversial, as these cells may contribute to both protection and pathology.^1,2^ It has been proposed that control of mycobacteria by neutrophils, especially virulent *M. tuberculosis*, demonstrates inter-individual variability,^3^ which may help to explain inter-individual differences in the ability of the innate immune system to control infection or dissemination, as well as differences in reported experimental results.^3,4^ However, techniques to assess growth restriction by human neutrophils are not well established. Assessment of neutrophil antimycobacterial activity using a classical colony-forming unit (CFU) assay has multiple limitations. First, cell-pathogen cultures are necessarily disturbed in the process of pathogen enumeration. Cell lysis to release organisms introduces new bio-active reagents and may be incomplete. Serial dilutions before plating CFU may be inaccurate, due to clumping and pipetting error, and the process is time-consuming. Furthermore, existing techniques for mycobacterial quantification (including radiometric assays such as Bactec^®^) require prolonged culture of organisms in growth medium after the end of the restriction assay. This not only increases the risk of contamination and represents a safety issue for the laboratory but also deviates the *in vitro* assay further from *in vivo* reality, where organisms would not have the opportunity to recover and replicate in the absence of immune challenge.

Another crucial aspect of host leucocyte function in addition to killing is phagocytosis of mycobacteria: this is either a prerequisite for elimination of the organisms or an essential stage in disseminating viable organisms to distant sites. However, assessment of this process is also challenging. Flow cytometric techniques^5–10^ avoid labour-intensive microscopy but suffer a number of potential pitfalls. Specifically, it has been little appreciated that vital dyes such as trypan blue, used to ‘quench’ extracellular fluorescence and to identify surface-bound organisms, also stain dead cells and will enter fixed cells.^7^ Furthermore, mycobacterial clumping in culture can significantly interfere with flow cytometry assays.^6^ Although various techniques can minimise this at the point of inoculation, the organisms tend to re-aggregate during incubation.^6^

Here we describe a novel, luminescence-based, in-tube assay of antimycobacterial activity for human neutrophils infected with either *Mycobacterium bovis* BCG (BCG) or *M. tuberculosis (M. tb)* together with a flow cytometric phagocytosis assay. These assays utilise mycobacteria whose bioluminescence is conferred by a plasmid encoding the AB segment of the *Vibrio harveyi* lux operon, as previously described.^11^ Light is emitted after addition of a substrate (1% n-decylaldehyde in ethanol), and this adenosine triphosphate (ATP)-dependent process reflects the metabolic activity of the organisms. Of note, transformation with this plasmid does not appear to negatively impact bacterial fitness or virulence, as previously demonstrated in an animal model.^11^

## 2 Materials and methods

### 2.1 Organisms and labelling

The plasmid construction and electroporation of organisms has been described previously.^11^ 1.5 ml vials of mycobacteria stored at −80 °C were defrosted and added to 15 mls (*M. tb*) or 20 mls (BCG) liquid 7H9 (Becton Dickinson)/ADC (Becton Dickinson) growth medium enriched with 0.05% Tween 80 (Sigma) and 1 mcl/ml hygromycin B (Roche diagnostics). Organisms were grown to mid-log phase (72 h) before use in these assays.

For Fluorescein isothiocyanate (FITC) labelling, 5 ml of mid-log phase organisms in 7H9 were centrifuged at 2000 × g for 5 min and resuspended in 1 ml carbonate-bicarbonate buffer (pH 9.6) containing 0.05% Tween 80. 5 mcl of FITC stock (Sigma), previously made to 100 mg/ml with Dimethyl sulfoxide (DMSO) and stored at −20 °C until use, was added and the suspension was incubated at 37 °C for 15 min. The labelled organisms were spun in a micro-centrifuge at 4000 × g for 2 min, the supernatant was aspirated and the pellet was resuspended in 1 ml Phosphate Buffered Saline (PBS) containing 0.05% Tween 80. This washing step was repeated twice and the organisms were then resuspended in 7H9.

Luminescence of stock was measured in a Berthold AutoLumat LB953 luminometer (for BCG) or in a Berthold Sirius single-tube luminometer (for *M. tb*, inside a Biosafety Cabinet) on duplicate samples of 100 mcl organisms added to 900 mcl PBS in 5 ml test tubes (Becton Dickinson). Both luminometers automatically inject 100 mcl of the substrate 1% n-decylaldehyde (Sigma) in ethanol.

The relative light unit (RLU) to colony forming unit (CFU) ratio for both stocks (established by contemporaneous RLU measurement and plating for CFU of mycobacteria growing in 7H9 medium) was determined to be approximately 3:1. Organisms were diluted in a standard volume of PBS to reach the required number of RLU for the experiments.

### 2.2 Neutrophil isolation

Human neutrophils were isolated either by magnetic beads or Percoll gradient from the peripheral blood of healthy consenting donors. For bead separation 4 ml freshly drawn heparinised blood was incubated for 15 min with 200 mcl magnetic human CD15 MicroBeads (Miltenyi Biotec) at 4 °C. During this time an LS column in a MidiMACS separation unit (Miltenyi Biotec) was ‘primed’ with 3 ml MACS buffer (0.5% bovine serum albumin + 20 mM Ethylene diamine tetra acetic acid (EDTA) in PBS). After incubation with beads the blood was diluted 1:1 with Roswell Park Memorial Institute-1640 medium (RPMI-1640) and pipetted onto the top of the LS column.

Once the blood had percolated through the column, 6 ml RPMI-1640 was added to wash out residual erythrocytes and loosely adherent cells. The column was then removed from the magnet and placed into a 15 ml Falcon tube, 2 ml RPMI-1640 was added to the top and plunged briskly through the column using the supplied syringe driver. The collected CD15 + cells were counted using a Beckman Coulter Ac.T Diff haematology analyser and diluted if necessary with further RPMI-1640 to reach the required final concentration.

For Percoll isolation, 30 ml heparinised blood was sedimented using 4 ml 6% Dextran (Sigma). The leucocyte-rich upper layer was then aspirated, transferred to a new 50 ml Falcon tube and centrifuged at 350 × g for 6 min. Pelleted cells were resuspended in 3 ml 55% Percoll and layered onto a discontinuous gradient of 3 ml 81% Percoll and 3 ml 70% Percoll. The gradient was centrifuged at 700 × g for 20 min with no deceleration. Granulocytes were harvested from the 71/80 interface.^12,13^ Both techniques consistently yield >95% purity granulocytes by Coulter counting.

### 2.3 Serum generation, inactivation and pre-opsonisation

Freshly drawn non-anticoagulated blood was centrifuged in a 15 ml Falcon tube at 500 × g for 15 min with minimal deceleration. Separated plasma was transferred to another Falcon tube and incubated in a water bath at 37 °C. After the platelets had plugged serum was aspirated for use.

Heat inactivation of serum was performed in a water bath at 56 °C for 30 min or at 90 °C for 2 min, as indicated. Heat inactivated fetal calf serum was obtained from Biochrom AG (Berlin, Germany).

For pre-opsonisation, 200 mcl BCG-lux suspension in 7H9 at a concentration of 5 million RLU (1.7 million CFU)/100 mcl was incubated at a 1:1 volume ratio with autologous serum for 20 min at 37 °C.

### 2.4 Mycobacterial restriction assay

Granulocytes were resuspended to an appropriate concentration (usually 1 × 10^6^ cells/ml) in RPMI-1640. 400 mcl of cell suspension was pipetted into a 5 ml Falcon flow cytometry tube (Becton Dickinson). 50 mcl autologous serum was added (giving a final concentration of 10% serum) followed by 50 mcl organism suspension, appropriately diluted to reach the required multiplicity of infection (MOI). The tubes were capped and then rolled to ensure that all organisms were mixed with the cell suspension before being incubated on their sides on a rocking plate (20 revolutions per minute (rpm)) at 37 °C. After the required time had elapsed, samples were allowed to cool to room temperature for five minutes (*V. harveyi* luciferase-induced luminescence is maximal at room temperature and relatively inhibited at 37 °C^14^), briefly vortexed, caps were removed and the tubes were placed in a luminometer for measurement.

### 2.5 Lysis

To lyse human cells, 1 ml 0.1% Saponin was added, samples were vortexed, incubated for 30 min and vortexed again. 1 ml PBS was added to control samples, which were otherwise treated identically. Permeabilisation was confirmed by microscopy of cells stained with trypan blue and by flow cytometry after addition of propidium iodide (see below).

### 2.6 Phagocytosis assay

500 mcl of CD15-positive granulocytes in RPMI-1640 at a concentration of 1 × 10^6^/ml were aliquotted into sterile 5 ml flow cytometry tubes. A suspension of organisms (pre-opsonised with autologous serum or non-opsonised) was added at an appropriate volume to reach the required MOI for the relevant experiment. The MOIs investigated, expressed as RLU:cells, were 10:1, 1:1, 1:2 and 1:10. These MOIs approximately equate to CFU:cell ratios of 3:1, 1:3, 1:6 and 1:30 respectively. Non-pre-opsonised samples had serum (or PBS in serum-free experiments) added at the same time as organisms and final volumes were adjusted with relevant media to ensure comparability of conditions.

Samples were capped and then rolled to ensure that all organisms were mixed with the cell suspension before being incubated on their sides on a rocking plate (20 rpm) at 37 °C for 30 min. During this time the centrifuge was cooled to 0 °C. After 30 min samples were placed immediately on ice for 5 min and then spun at 300 × g for 5 min in the pre-chilled centrifuge. Samples were returned to ice and supernatants were aspirated, leaving approximately 100 mcl volume. 1 mcl eFluor450 Fixable Viability Dye (eBiosciences) and 0.5 mcl Phycoerythrin (PE)-conjugated anti-CD66a, c, e antibody was added to the pellets and samples were incubated for 12 min. Subsequently 1 ml of ice-cold PBS and 12.5 mcl of 0.2 micron syringe-filtered Trypan blue (Sigma) was added to the tubes and they were transferred back to the centrifuge at 0 °C. (Note that syringe filtering of trypan blue is required because it becomes highly particulate in suspension and can interfere with flow cytometry.) After a further centrifuge spin at 300 × g for 5 min, supernatants were aspirated and the pellets were resuspended in 500 mcl 2% paraformaldehyde (BCG) or 4% paraformaldehyde (*M. tb*).

### 2.7 Flow cytometry

Flow cytometry was performed immediately (BCG samples) or the following day on a Becton Dickinson Fortessa machine. Voltages were used as follows: Forward Scatter (FSC) (Height & Area) = 280, Side Scatter (SSC) = 258, Allophycocyanin (APC) = 604, Pacific Blue = 467, Alexa Fluor 488 = 511, PE = 450. The threshold set on Forward Scatter Area was 24,667. A total of 50,000 events were collected for each cell sample.

### 2.8 Assessment of clumping

To assess the extent of clumping of mycobacteria in the phagocytosis experiments, parallel samples were processed without human cells. Volumes of organisms and serum were identical, but 500 mcl RPMI-1640 was used instead of a granulocyte suspension. The samples were acquired on the flow cytometer for the maximum duration taken by a contemporaneous cell sample to reach 50,000 events.

### 2.9 Compensation controls

An identically processed aliquot of uninfected cells and serum was stained with CD66a, c, e-PE alone. Another aliquot of cells was divided into two and one half was heat-shocked at 60 °C in a water bath for 20 min. The two halves were then recombined, mixed and separated into two again. One of these was labelled with eFluor450 Fixable Viability Dye and the other with trypan blue. A further sample of granulocytes contained organisms only with no dyes or fluorochromes and a final control contained neither organisms nor fluorescent molecules.

### 2.10 Analysis

Analysis was performed with FlowJo software Version 7.6.1 (Treestar). Compensation parameters were derived from the single stained and unstained samples and applied to the experimental samples. To analyse experimental samples, first doublet signals were excluded by plotting Forward Scatter Area versus Forward Scatter Height. Subsequently dead cells were excluded on a plot of eFluor450 Viability Dye versus APC (trypan blue signal). Neutrophils were then gated within the live cells as CD66a, c, e-PE positive events with high side scatter. These were divided into quadrants by plotting FITC versus APC (emission spectrum of trypan blue).

### 2.11 Confocal microscopy

Samples were incubated using the same reagent quantities as described above in 8-chambered coverslips (Lab-Tek) and images were obtained on live cells using a Zeiss Axiovert LSM 510 Meta NLO Confocal Microscope. Nuclei were stained using the membrane permeable dye Hoechst 33342 (Anaspec). Some experiments were undertaken using Green Fluorescent Protein (GFP)-expressing BCG-lux organisms, in which GFP is encoded on the same plasmid as the lux AB. Image z-stacks were acquired using the 488 nm laser line at 5% transmission for excitation of GFP and differential interference contrast, and two-photon excitation of Hoechst 33342 with a Spectra-Physics MaiTai DeepSee laser set to 1% transmission at 750 nm. GFP/FITC fluorescence was detected using a 505–530 nm, and Hoechst 33342 with a 420–460 nm bandpass filter. Image z-stacks were rendered into orthogonal projections to demonstrate internalization of BCG.

### 2.12 Statistics

Comparison of two groups was performed by two-tailed paired Student's *t*-test. Comparison of several groups was performed by one-way analysis of variance (ANOVA) with post-hoc Bonferroni correction for parametric data or by Kruskal–Wallis test with post-hoc Dunn's correction for non-parametric data. All statistics were performed using GraphPad Prism Version 4.00.

## 3 Results

### 3.1 Neutrophils restrict bioluminescence of BCG-lux and *M. tb*-lux in a dose-dependent manner

**Figure 1:**
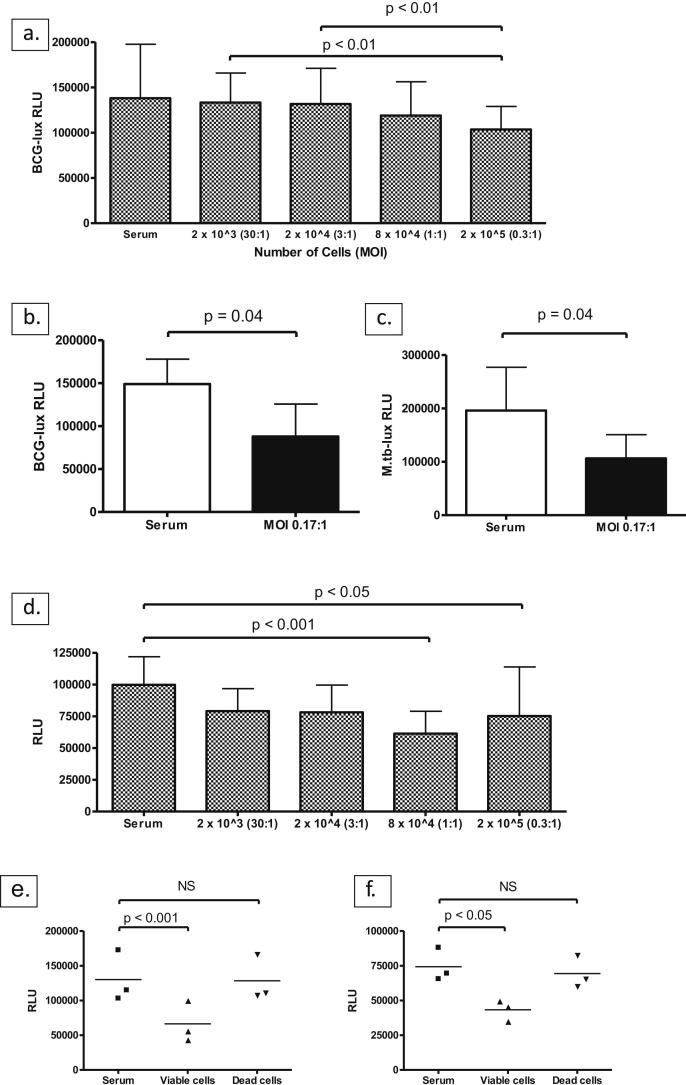
Restriction of mycobacterial luminescence by neutrophils is inversely proportional to multiplicity of infection and requires viable cells. a. Luminescence of BCG-lux (Relative Light Units, RLU) at one hour post-inoculation according to MOI. Column heights represent the mean results from nine separate donors (neutrophils isolated by MicroBeads) performed in triplicate for each MOI; error bars indicate standard deviation (SD). The inoculum was standardised to 200,000 RLU (80,000 CFU) and the number of cells was varied as indicated. The serum control contained no neutrophils. Overall *p*-value for one-way ANOVA < 0.0001. b. Luminescence of BCG-lux using higher ratio of neutrophils to organisms. Column heights represent the mean results from six different donors (neutrophils isolated by Percoll gradient) performed in triplicate for each donor; error bars indicate SD. Other experimental conditions as in (a). c. Luminescence of *M. tb*-lux using same MOI as in (b). Column heights represent mean results from four separate donors (neutrophils isolated by MicroBeads) performed in triplicate for each donor; error bars indicate SD. Other experimental conditions as in (a). d. 24 h luminescence readings from same experiments presented in (a). Overall *p*-value for one-way ANOVA < 0.0001. e. and f. Luminescence of BCG-lux (200,000 RLU/80,000 CFU inoculum) incubated in RPMI-1640 with either 10% serum only (‘Serum’), serum plus viable neutrophils at MOI 0.17:1 (‘Viable cells’) or serum plus neutrophils pre-heat-shocked at 60 °C for 20 min at MOI 0.17:1 (‘Dead cells’); luminescence was measured at 1 h (e) or 24 h (f). Markers represent the mean of triplicate readings per condition (3 donors).

Isolated neutrophils from nine donors were infected with a fixed inoculum of BCG-lux across a variety of neutrophil numbers. Luminescence measured at one hour post-inoculation showed a clear negative correlation with the number of neutrophils (Figure 1a). We repeated the experiment using six separate donors and a higher ratio of neutrophils to organisms: this demonstrated a greater reduction in recovered luminescence than was seen at higher MOI (Figure 1b). The degree of suppression of bioluminescence using an MOI of 0.17 was similar for *M. tb*-lux (cell-containing samples exhibited mean 57.8% [range 41.8%–87.3%] luminescence of serum-only samples, see Figure 1c) and BCG-lux (cell-containing samples exhibited mean 62.1% [range 32.7%–105.4%] luminescence of serum-only samples, see Figure 1b). At 24 h, the ability of cells to restrict mycobacterial luminescence versus serum was more significant than at one hour (Figure 1d), although interestingly there was relatively less effect at the highest MOI by this time point. On the basis of these results we suggest that an MOI of approximately 1 CFU:3–6 cells is optimal to demonstrate an appreciable cell effect and yield inter-individual variability. Of note, the addition of dead (heat-shocked) neutrophils to mycobacteria had no effect on mycobacterial bioluminescence at either 1 h or 24 h (Figure 1e and f).

### 3.2 Lysis of neutrophils is not required in these experiments for accurate measurement of antimycobacterial effects

**Figure 2:**
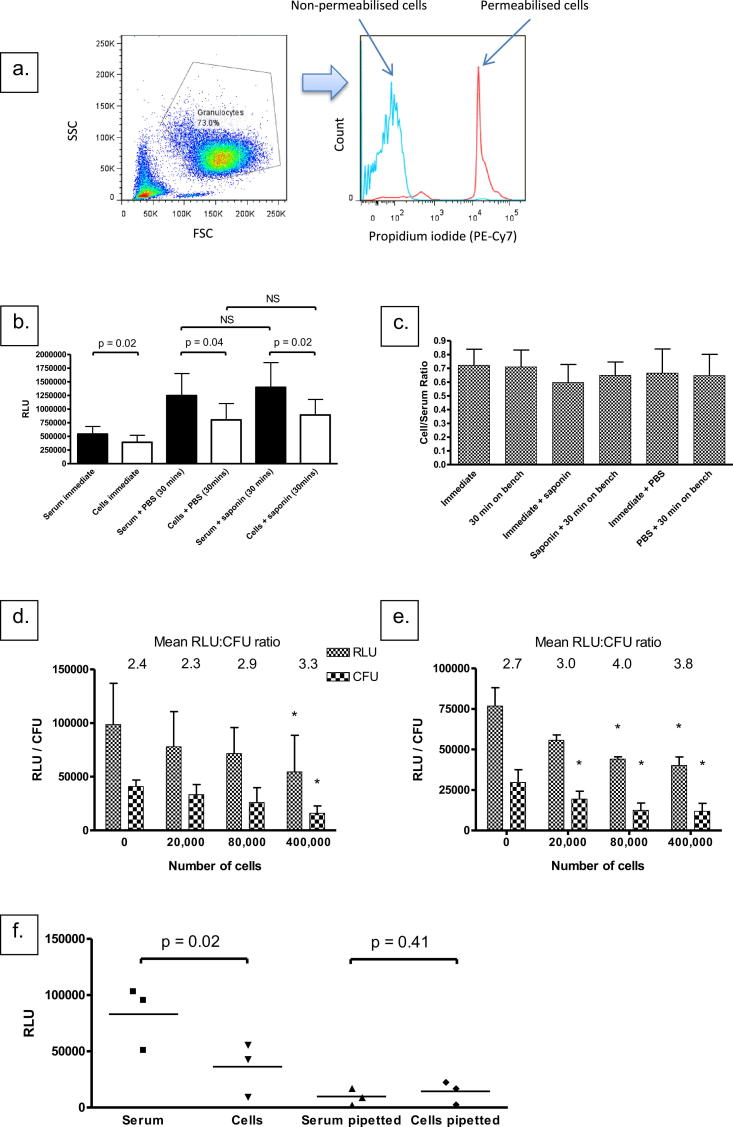
Lysis or washing of cells results in artefactual increases in luminescence only. a. Saponin treatment permeabilises neutrophils. Samples of 400,000 neutrophils in 10% autologous serum were treated with 1 ml 0.1% saponin (red line) or 1 ml PBS (blue line) for 30 min and then incubated with 5 mcl propidium iodide for 20 min before acquisition on the flow cytometer. Figure shows granulocyte gate as set by forward and side scatter and propidium iodide signal (detected in PE-Cy7 channel). b. Saponin appears to increases luminescence in cell samples, but this effect is mediated by PBS alone. 50 mcl BCG-lux (400,000 RLU (130,000 CFU)) was inoculated into either 400 mcl RPMI-1640 plus 50 mcl autologous serum (‘serum’) or 400 mcl neutrophil suspension (MOI = 1 CFU:3 neutrophils) in RPMI-1640 plus 50 mcl autologous serum (‘Cells’). After one hour's incubation samples were allowed to cool and either vortexed and placed immediately in the luminometer (‘immediate’) or had 1 ml 0.1% saponin added, vortexed and incubated for 30 min before measurement in the luminometer (‘saponin’) or had 1 ml PBS added, vortexed and incubated for 30 min before measurement in the luminometer (‘PBS’). Column heights represent mean values from four separate experiments, performed in triplicate at each occasion; error bars represent SD. *p*-values from paired *t*-tests. NS = Not significant. c. Ratio of cell luminescence to serum luminescence does not change depending on condition. Ratio of cell:serum luminescence was calculated on samples from (b), also including measurements on serum and cell samples immediately after the addition of PBS or saponin. *p* > 0.05 across all conditions. Column heights represent means, error bars represent SD. d and e. CFU show similar pattern to RLU readings. RLU (left-hand columns, small checks), and colony forming units (right-hand columns, large checks) in 500 mcl samples containing 10% autologous serum and the number of neutrophils as indicated on the *x*-axis, initially inoculated with 200,000 RLU (80,000 CFU) BCG-lux. RLU and CFU were measured after 1 h (d) or 24 h (e) incubation at 37 °C. Column heights represent means from two separate experiments, performed in triplicate for each condition; error bars represent SD. * = *p* < 0.05 versus serum control. Overall *p*-values for one-way ANOVA: 1 h RLU, 0.019; 1 h CFU, 0.019; 24 h RLU, 0.027; 24 h CFU, 0.010. Above each pair of columns is the mean RLU:CFU ratio for that MOI and time point. f. There is no greater residual luminescence in cell versus serum samples after replacing culture medium. Samples containing 200,000 RLU (80,000 CFU) BCG-lux, 10% autologous serum and either no neutrophils (‘Serum’) or 400,000 neutrophils (‘Cells’) were incubated at 37 °C for one hour. Sample luminescence was either measured directly or culture medium was pipetted away and 500 mcl fresh RPMI-1640 added before measurement (‘Serum pipetted’ and ‘Cells pipetted’). Results represent three separate experiments, performed in duplicate for each condition; *p*-values derived from paired *t*-tests.

Although the substrate for the lux construct is an aldehyde, which would be expected to diffuse freely through cell membranes,^15^ it could not be assumed that this would reach bacilli inside the phagosome of viable neutrophils. It was important to establish that the assay did indeed measure both intracellular and extracellular bacilli, since neutrophil killing may occur either inside or outside the cell. We thus proceeded to establish whether neutrophil lysis influenced measurement of mycobacterial bioluminescence, and hence accurate estimation of the antimycobacterial effect of neutrophils, in these experiments. Lysis using 0.1% saponin solution in PBS, which permeabilised all human cells to propidium iodide (Figure 2a), did not have a different effect on mycobacterial luminescence to the addition of PBS vehicle alone (Figure 2b). An apparent increase in luminescence in both PBS and saponin-treated samples largely occurred immediately, most likely due to the increased volume (which perhaps increased the amount of sample exposed to the luminometer's light sensor). However, the ratio of luminescence in samples containing cells to that in samples containing serum alone was not influenced by the addition of saponin solution vs. PBS vehicle control (Figure 2c). We therefore maintained the assay volume at 500 mcl to ensure an adequate density of neutrophils in the assay, which may be limited depending on isolation method or blood volume. Plating for CFU of lysed samples also revealed the same pattern as RLU data (Figure 2d and e), suggesting that luminescence results indicating mycobacterial restriction by neutrophils are accurate. There was good correlation between RLU and CFU: at 1 h Pearson *r* = 0.88 (95% CI 0.46–0.98, *p* = 0.004); at 24 h Pearson *r* = 0.95 (95% CI 0.74–0.99, *p* = 0.0003).

We also investigated the time required to reach maximum luminescence in cell-containing and cell-free samples. There was a longer delay before peak luminescence in the cell samples, consistent with diffusion of the substrate across additional membranes (Supplementary Figure 1a). Prolonged measurement times do not ‘close the gap’ between the cell samples and the serum-only samples, suggesting that there is not a slow or delayed diffusion of substrate to viable intra-cellular organisms (Supplementary Figure 1b). We therefore conclude that 20 s is sufficient measurement time.

Finally, the difference in luminescence between serum-only and cell-containing samples persists to 24 h (Figure 1d), a time point when most of the neutrophils have undergone cell death. In experiments using BCG-lux with MOI of 1 CFU:3 cells, cell-containing samples exhibited a mean of 81.6% (±26.6%) of the luminescence of corresponding serum-only samples by one hour (across nine donors). At 24 h this figure was 73.7% (±28.5%), confirming that the organisms do not recover after neutrophil death and hence that cell-mediated reduction in luminescence does not only reflect early phagocytosis (as noted earlier, dead cells do not directly impact upon mycobacterial luminescence).

### 3.3 Neutrophils are not lost due to adherence to walls of luminometer tubes

Early experiments confirmed that without rocking samples during incubation there was rapid neutrophil cell death and failure to eliminate virulent mycobacteria, consistent with recent reports.^4^ However, lying tubes horizontally raised the concern that adherence of neutrophils to the walls of the tubes during incubation could lead to cells ‘sequestering’ bacilli away from the light sensor in the luminometer. An investigator blinded to time point measured total sample volume and performed microscopy with counting chambers on aliquots of vortexed samples before and after one hour's incubation. Volumes did not decrease and, across a range of cell densities, we did not observe any significant reduction in the number of neutrophils counted by one hour (mean difference in cell count before and after incubation across four experiments = 0.4%; *p* = 0.96).

We continued to investigate this potential issue by pipetting away the culture medium after one hour's incubation and replacing with fresh medium. As demonstrated in Figure 2f, there was no greater residual luminescence in cell-containing samples vs. serum-only samples: again, this suggests that bacilli are not sequestered in adherent cells.

### 3.4 The phagocytosis assay demonstrates clear populations of neutrophils defined by the presence and location of organisms

**Figure 3:**
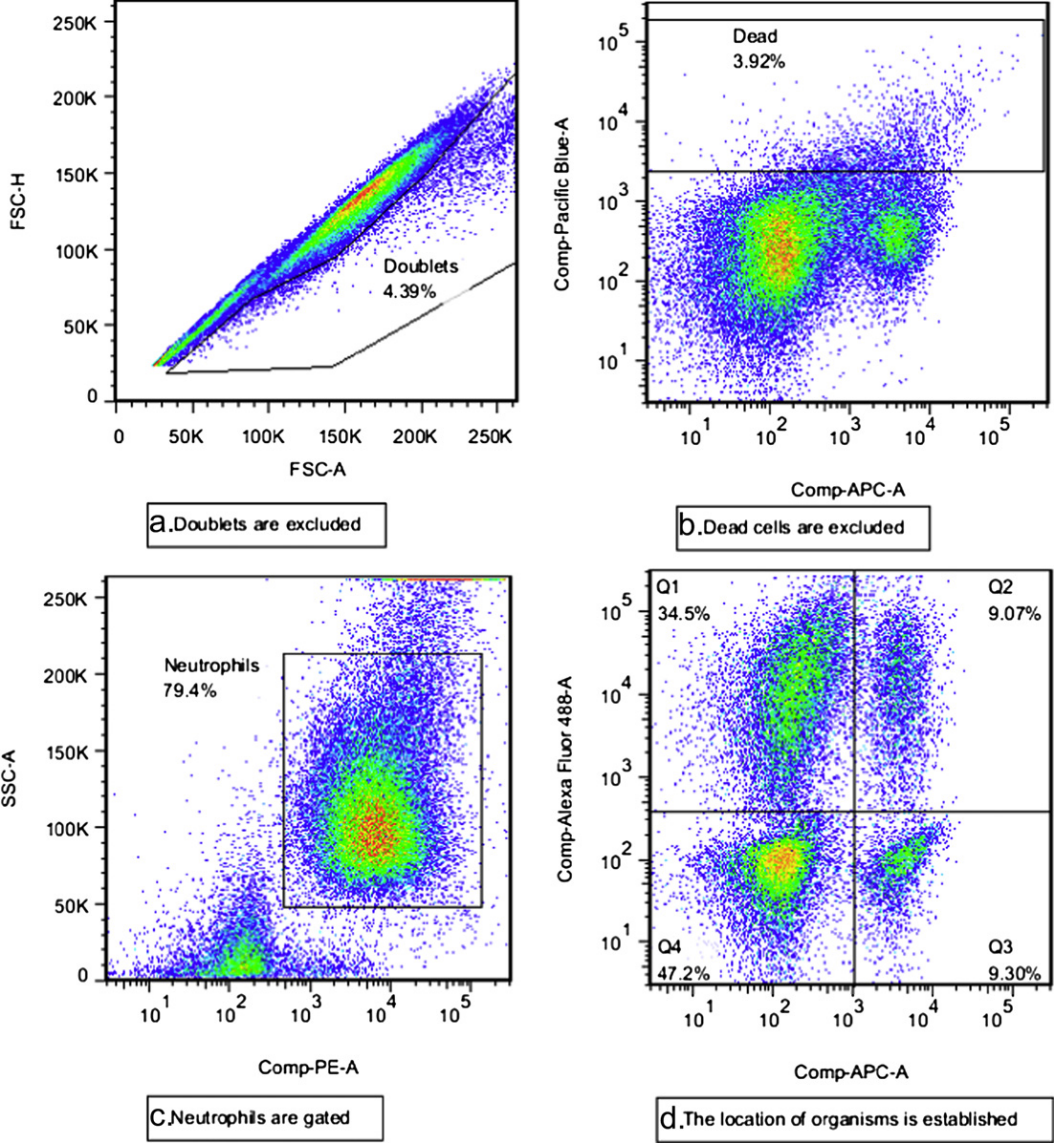
Gating strategy and interpretation for phagocytosis assay. a. First, doublet signals are excluded by plotting forward scatter area versus forward scatter height. b. Dead cells are excluded on the basis of positivity for eFluor450 Fixable viability dye (signal seen in Pacific Blue channel); note that most dead cells are also positive for trypan blue (signal seen in APC channel). c. Neutrophils are defined as positive for PE-conjugated CD66a, c, e and high side scatter. d. Neutrophils are divided into quadrants on the basis of trypan blue signal (APC) and FITC signal (Alexa Fluor 488). Q1 (FITC positive, trypan blue negative): Internalised organisms only; Q2 (FITC positive, trypan blue positive): Internal and external organisms; Q3 (FITC negative, trypan blue positive): External organisms only; Q4: (FITC negative, trypan blue negative): not associated with organisms.

Figure 3a–d demonstrate that when using BCG-lux, the phagocytosis assay clearly identifies cells with intracellular and extracellular organisms. FITC positive, trypan blue negative cells have internalised organisms only; FITC positive, trypan blue positive cells have both internal and external organisms; FITC negative, trypan blue positive cells only possess external organisms while dual negative cells are not associated with organisms.

### 3.5 Significant clumping of organisms does not occur at lower organism concentrations

Since mycobacteria clump in culture, we investigated whether this phenomenon may interfere with our experiments. Samples of FITC-labelled organisms with autologous serum in RPMI were processed identically to cell samples from six donors and then acquired on the flow cytometer for the maximum duration taken by a contemporaneous cell sample to reach 50,000 events.

**Figure 4:**
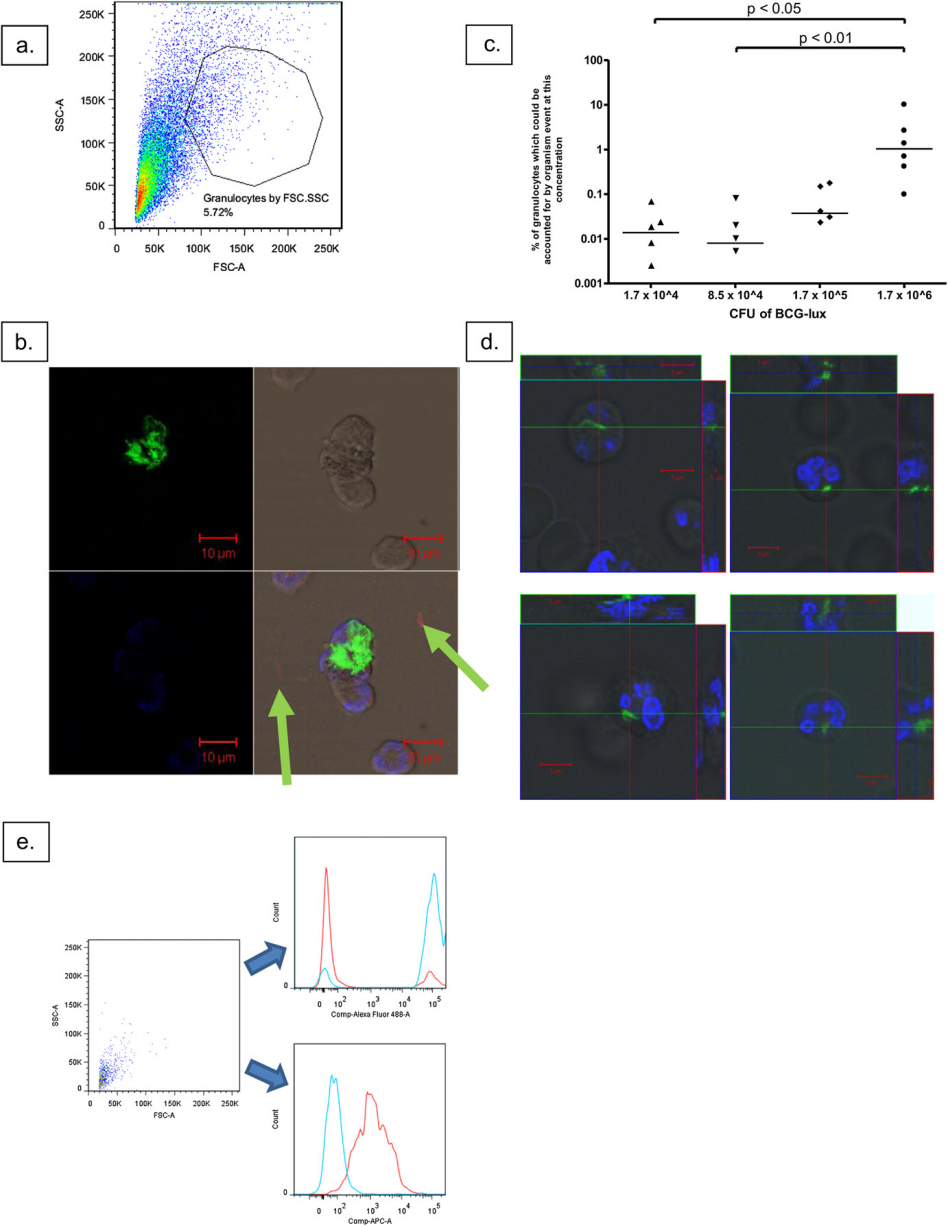
Assessment of clumping. a. A Forward Scatter versus Side Scatter plot of BCG-lux organisms (with 10% serum in RPMI-1640) demonstrates how organisms can mimic cells; the granulocyte gate was derived from a contemporaneous cell sample from the donor of the serum. b. Confocal microscopy image of a clump of BCG organisms attached to a neutrophil; note that the green fluorescence of individual external organisms has been ‘quenched’ by trypan blue and they now fluoresce red (arrows), while the clump of organisms remains green. Nuclei are stained with Hoechst 33342. c. Samples of BCG-lux at different concentrations in the presence of human serum in RPMI-1640 were processed identically to cell-containing samples and results acquired on the flow cytometer. The number of events in these organism-only samples seen inside a granulocyte gate derived from a contemporaneous cell sample (on the basis of forward and side scatter) are here expressed as a percentage of the number of viable neutrophils in that cell sample; note the logarithmic y-axis and hence results are not shown if they are zero. Each marker represents one donor; lines represent medians. Overall *p*-value for Kruskal-Wallis test = 0.0028. d. Orthogonal Confocal microscopy images confirm in three dimensions that organisms (here GFP-expressing BCG-lux) are internalised and, as it typical at an MOI of 1 CFU:6 cells, most neutrophils only contain one or two organisms. Nuclei are stained with Hoechst 33342. e. Samples of 8 × 10^4^ CFU FITC-labelled BCG-lux were prepared in 1 ml PBS with 12.5 mcl trypan blue (red line) or without trypan blue (blue line), centrifuged and fixed in 2% paraformaldehyde before acquisition on the flow cytometer. Signal in the Alexa-Fluor 488 (FITC) and APC (trypan blue) channels is represented for both samples; trypan blue treatment results in near-total loss of FITC signal and increase in APC signal.

Figure 4a shows that at high organism concentrations (5 × 10^6^ RLU/1.7 × 10^6^ CFU in a 500 mcl sample, the concentration used to create MOI 3 CFU:1 cell in these experiments), clumping can be significant and that organisms may mimic granulocytes by forward and side scatter. Although a cell surface marker may minimise this problem, Figure 4b shows how a clump of organisms attached to a cell (hence surface marker positive) may retain its green signal even after ‘quenching’ with trypan blue. However, at lower concentrations clumping of organisms is not a significant problem, with ‘granulocyte-mimicking’ organism events representing less than 0.1 percent of true cell events (Figure 4c). Confocal imaging confirmed that at lower MOIs most neutrophils contained only one or two bacilli (Figure 4d). We also confirmed that at this concentration of organisms trypan blue is able to quench FITC fluorescence and lend red fluorescence to organisms (Figure 4e).

### 3.6 Internalisation increases with MOI, but not in a linear fashion

**Figure 5:**
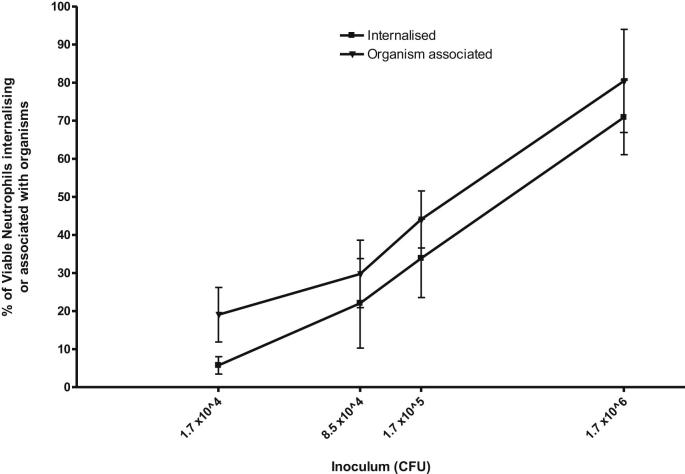
Determining optimal multiplicity of infection to demonstrate phagocytosis. Percentage of viable neutrophils with internalised organisms or ‘organism associated’ (includes those cells with external organisms only) according to infecting inoculum of pre-opsonised FITC-labelled BCG-lux. The *x*-axis is a log_10_ scale, and inocula used for these experiments are indicated. Expressed as MOI (CFU:cells), 1.7 × 10^4^ CFU = 1:30; 8 × 10^4^ CFU = 1:6; 1.7 × 10^5^ CFU = 1:3; 1.7 × 10^6^ RLU = 3:1. Markers represent mean of six experiments, error bars represent standard deviation. Neutrophils isolated by MicroBeads.

An analysis of multiplicity of infection is shown in Figure 5 and Table 1. As can be seen, there was an approximately linear increase in both internalisation and total percentage of cells which are ‘organism associated’ (i.e. those with both internalised and external organisms) up to an MOI of 1 CFU:3 cells. After this the increase plateaus.

At the lowest MOI (1 CFU:30 cells), there was less heterogeneity between donors and more external binding. At the highest MOI (3 CFU:1 cell) the forward and side scatter properties of the cells were altered and the number of viable neutrophils counted per 50,000 events was significantly reduced (data not shown). Use of MOI of 3 CFU:1 cell was also associated with a very high FITC median fluorescence intensity (MFI) which interfered with the PE signal of the neutrophil cell surface marker CD66a, c, e even after compensation.

We therefore utilised an MOI of 1 CFU to 3–6 cells for optimal results in terms of both minimising organism clumping (Figure 4) and easily measuring internalisation. These MOIs are also suitable for the restriction assay (see above).

We were able to repeat the assay in three of the donors, maintaining MOI 1:3. The mean difference in percentage of neutrophils internalising mycobacteria between the first and second experiments was only 1.76%, suggesting good reproducibility of the method.

### 3.7 Internalisation is inhibited by low temperature and results are comparable with those using acid-resistant fluorophores

As a test of the reliability of the assay, we processed matched samples incubated at either 37 °C or on ice (Supplementary Figure 2). Ice inhibited internalisation significantly (using MOI 0.3 CFU:1 cell, mean percentage of neutrophils with internalised organisms at 37 °*C* = 39.2% ±11.3%, mean percentage of neutrophils with internalised organisms at 0 °*C* = 2.8% ±3.1%, *p* = 0.047) There was also a trend towards greater external binding when incubated on ice (using MOI 0.3 CFU:1 cell, mean percentage of neutrophils with external binding at 37 °*C* = 9.9% ±7.1%, mean percentage of neutrophils with external binding at 0 °*C* = 19.4% ±13.0%).

Due to concerns regarding the acid resistance of FITC, we also compared the assay with experiments using the acid-resistant fluorophore pHrodo™ (Invitrogen): Supplementary Figure 3 details the experimental procedure and gating strategy. The mean internalisation using BCG-lux and an MOI of 0.3 was 37.6% using pHrodo™ versus 35.5% for our assay, and we thus consider results to be comparable.

### 3.8 Trypan blue stains dead and fixed cells

**Figure 6:**
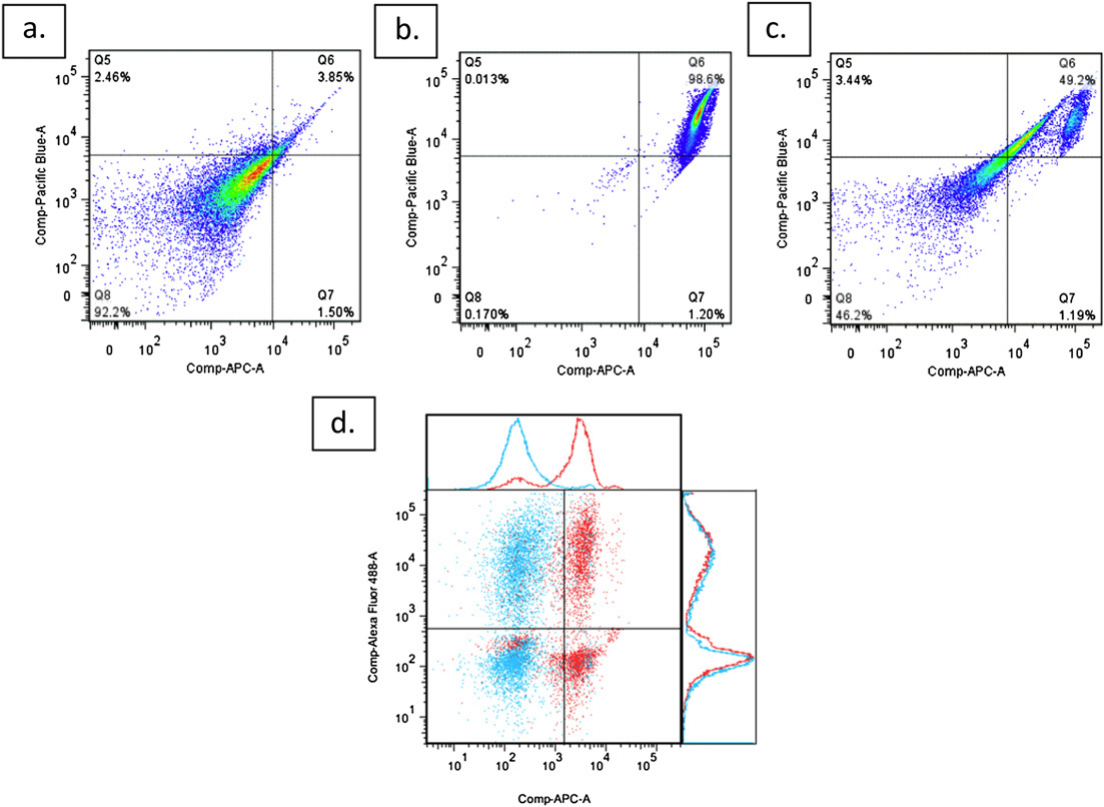
Trypan blue stains dead and prolonged fixed cells. a–c. Concordance of eFluor450 Fixable viability dye (fluorescence detected in the Pacific Blue channel) and trypan blue (fluorescence detected in APC channel). Samples of 400,000 neutrophils without organisms were stained with 12.5 mcl trypan blue and 1 mcl eFluor450 Fixable viability dye only; a – freshly isolated cells; b – cells heat-shocked at 60 °C for 20 min; c – sample containing half freshly isolated and half heat-shocked cells. Plots include all singlet events. d. A sample of neutrophils containing FITC-labelled BCG organisms was acquired immediately (blue) and the following day (red); note the shift on the trypan blue axis (APC channel) but little change in the FITC (Alexa-Fluor 488) axis.

As mentioned above, trypan blue is commonly used in flow cytometry based phagocytosis assays to ‘quench’ extracellular fluorescence, and since it fluoresces itself in the red spectrum can be used to identify surface-bound organisms.^8,10^ However, trypan blue also enters dead cells and these could therefore be interpreted as organism-associated events. Samples processed without organisms showed good concordance between trypan blue and a Fixable Viability Dye (eFluor450) in identifying dead cells: see Figure 6a–c.

Using a Viability Dye in our experimental samples excluded a mean of 1097 (±407) events per sample, which represented 3.77% (±0.11%) of viable neutrophils.

We were also concerned that fixed cells would become positive for trypan blue signal. To investigate this, samples with a 1 CFU:3 cells MOI were processed again the following day. After this prolonged fixation (17–24 h) nearly all events had become positive for trypan blue (see Figure 6b). However, there was only limited quenching of internalised signal: the mean percentage of FITC positive events on the following day was 95.7% that of the immediately acquired samples.

### 3.9 Non-heat inactivated autologous serum is optimal to demonstrate significant phagocytosis and mycobacterial restriction

Phagocytosis of mycobacteria by neutrophils is likely to be influenced significantly by opsonisation^16^ and we therefore investigated the need for pre-opsonisation and compared autologous and fetal calf serum as opsonins.

**Figure 7:**
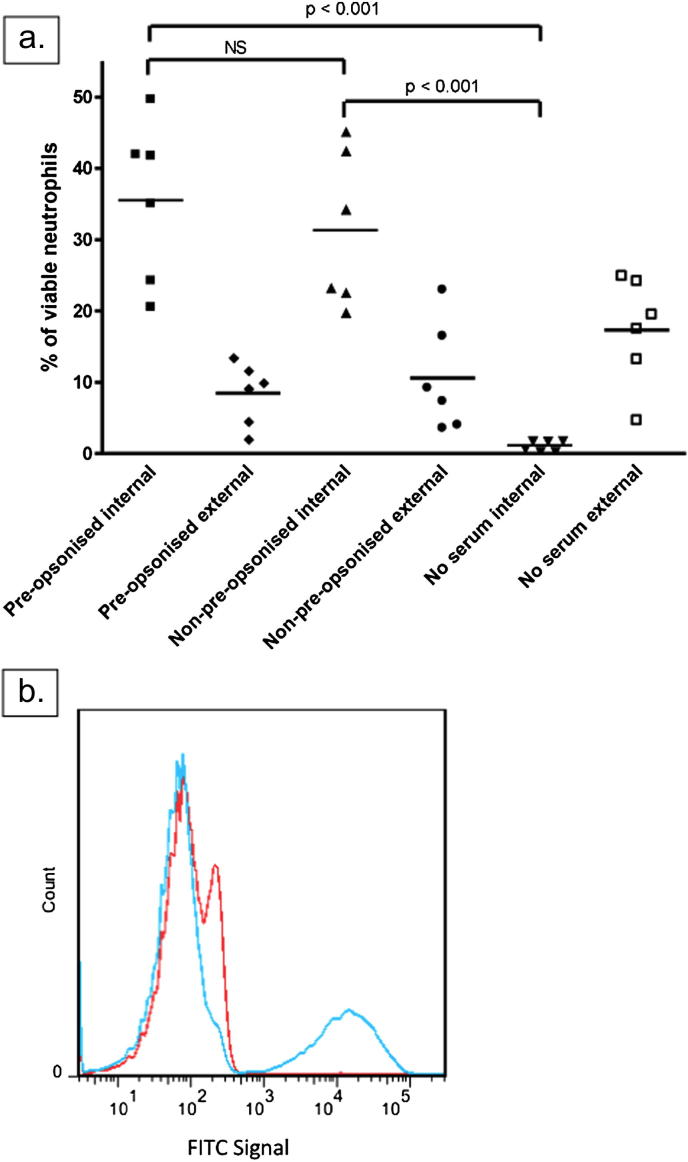
Investigation of opsonisation. a. Internalisation and external binding of FITC-labelled BCG-lux by viable MicroBead-isolated neutrophils according to opsonisation. All conditions performed with MOI 1 CFU:3 cells and incubated for 30 min. ‘Pre-opsonised’ – organisms were incubated with autologous serum for 20 min at 37 °C before infection of cells. ‘Non-pre-opsonised’ – organisms and serum were added at the same time. ‘No serum’ – organisms were added without serum. Each marker represents one donor; lines represent means. Overall *p*-value for one-way ANOVA < 0.0001. b. Plot of FITC signal in viable neutrophils from one donor incubated (1 CFU:3 cells) with labelled organisms for 30 min. Blue line: organisms pre-opsonised for 20 min with autologous serum; red line: organisms pre-opsonised for 20 min with heat inactivated fetal calf serum (contemporaneous sample).

Using an MOI of 1 CFU:3 cells we processed contemporaneous samples either pre-opsonised (20 min incubation of organisms and serum), non-pre-opsonised but with serum added at the same time as organisms, or no serum at all. Results of internalisation and external binding for six donors are shown in Figure 7a. There was no significant difference between the percentages of neutrophils internalising organisms when pre-opsonised or when serum was added at the same time as mycobacteria. However, there was a highly significant difference (*p* < 0.001) between both these conditions and the percentage of neutrophils internalising organisms in the ‘no serum’ samples. There was a non-significant trend towards greater external binding in the absence of serum.

Since the results of this assay reflect both the donor's cellular phagocytic capability and the extent of opsonisation we repeated the assay, using an MOI of 1 CFU:3 cells, with stored serum from all six donors but a single neutrophil donor. In terms of total internalisation, the coefficient of variation between donors reduced from 30.5% when using autologous neutrophils to 12.5% when using a single neutrophil donor.

Heat inactivation of serum at 56 °C reduced internalisation (by the same single donor's neutrophils) significantly but not entirely: mean value reduced from 47.7% to 7.9% of neutrophils internalising. However, the coefficient of variation among the heat inactivated serum samples was higher than with intact serum, at 36.1%.

Use of Heat inactivated Fetal Calf Serum yielded similar results to the ‘No serum’ condition (see Figure 7b), with internalisation less than 1%.

Non-heat-inactivated serum was also optimal for sustaining mycobacterial luminescence in the restriction assay, and hence allowed maximum demonstration of neutrophil-mediated restriction. RPMI alone significantly reduced mycobacterial bioluminescence (see Supplementary Figure 4). Heat inactivation of serum at 56 °C, and even more so at 90 °C, reduced mycobacterial luminescence towards the level seen in RPMI alone, and this pattern was maintained to 24 h. The use of Hanks' Balanced Salt Solution (HBSS) resulted in proportionally higher readings for all conditions but the same pattern of results was maintained (data not shown).

## 4 Discussion

We have here demonstrated a novel, simple technique to assess restriction of mycobacteria by human neutrophils, and optimised a phagocytosis assay which can be used contemporaneously.

The restriction experiments are performed in single tubes without transferring or disturbing the medium during analysis; extensive evidence confirms that lysis of human cells is not required. As expected, increasing the ratio of neutrophils to mycobacteria improves restriction on average, but there is considerable variability depending on donor – as previously proposed.^3^ This heterogeneity suggests that the technique may prove useful in detecting important differences in innate immune function.

Luminescent organisms carry a number of advantages for these assays: the ability to accurately standardise inocula (vital if performing cross-sectional inter-donor or prospective intra-donor analysis), the capacity to perform high throughput experiments with multiple replicates of experimental conditions, and instant results. It should be noted that the ‘read-out’ in the restriction experiments is a marker of mycobacterial metabolism and, although there does appear to be good correlation with CFU results (Figure 2d and e), the assay does not directly measure bacterial numbers. Nevertheless, it is arguable that luminescence is a better marker of overall organism ‘fitness’,^11^ and since there is no recovery of luminescence over 24 h despite death of the granulocytes we can conclude that the impact on fitness by neutrophils is long-lived. Neutrophils do not of course work in isolation in the body and, as ‘first responders’ before the arrival of other cells, a substantial impact on mycobacterial health, rendering organisms more susceptible to killing, may be as significant as killing itself.

The phagocytosis assay presented here resolves a number of issues of particular concern for experiments using mycobacteria and neutrophils. First, if the density of organisms is too high then clumping can be significant enough to interfere with flow cytometric analysis. However, we have discovered that at lower concentrations (below approximately 5 × 10^5^ RLU, or 1–2 × 10^5^ CFU, in 500 mcl total volume) this issue is not significant. At lower MOI it is therefore not essential to use a cell surface marker to confirm that a ‘granulocyte’ event, as defined by forward and side scatter, is a mammalian cell. Nevertheless, we include CD66a, c, e -PE in our assay since it also serves as a useful marker of neutrophil activation and degranulation.

Conversely, too low a concentration of organisms (5 × 10^4^ RLU, or 1.7 × 10^4^ CFU incubated with 4 × 10^5^ neutrophils in 500 mcl volume) reduces inter-donor variability and appears to increase external binding. This latter finding may relate to a ‘delay’ in the neutrophils encountering the organisms.

We also investigated the correct usage of the quenching agent trypan blue in this assay. Since neutrophils die rapidly *in vitro*, a significant percentage of cell events may be dead and hence artefactually trypan blue positive: it is therefore essential to also use a Viability Dye. It should be noted that the emission spectrum of trypan blue is broad in the red and far-red spectrum. We found similarly high MFI for trypan blue positive events among single-stained control samples in APC, Phycoerythrin-cyanin 5.5 (PE-Cy5.5), PE-Cy7 and Peridinin Chlorophyll Protein-Cyanin 5.5 (PerCP-Cy5.5). This necessarily limits the number of extra fluorochromes which can be added to the assay.

Another important aspect is the need for prolonged fixation to ensure death of organisms when using virulent mycobacteria. As we have demonstrated, this permeabilises cells to trypan blue and as such the phagocytosis assay cannot be effectively used to resolve surface-bound organisms with *M. tb*. Importantly however, internalised signal does remain fairly reliable.

Despite these issues, we still believe that a quenching agent is the best solution to prove internalisation compared to other strategies. Phagocytosis inhibitors (eg cytochalasin D) to create a ‘negative’ control^17^ may not completely abolish internalisation and may result in greater relative surface adherence; incubating samples on ice appears to induce the same phenomenon (Supplementary Figure 2). Use of a secondary labelling antibody to identify mycobacteria on cell surfaces and removing these events (9) will also remove signals from cells which have both internalised and adherent organisms. Antibiotic elimination of extracellular organisms is unlikely to occur rapidly enough to allow processing of samples before neutrophil cell death, and fluorochrome labels can remain fluorescent on dead organisms. The pH-sensitive fluorochrome pHrodo™ (Invitrogen) only fluoresces strongly when the pH of its micro-environment falls, consistent with internalisation into the phagosome. However, this does not enable resolution of surface-bound organisms, virulent mycobacteria may prevent deacidification of the phagosome,^18^ and we found that the labelling procedure significantly affected viability resulting in difficulty with standardising inocula and consequent variability in the assay.

We also investigated the impact of opsonisation on neutrophil phagocytosis of mycobacteria. Although a separate period of pre-opsonisation was not required, serum was clearly needed to permit internalisation (see Figure 7). Heat inactivation significantly reduced the percentage of neutrophils internalising organisms, perhaps suggesting an important role for complement in this regard. It was interesting to note that the assay worked successfully using a single donor's neutrophils but different donors' serum, allowing us to directly compare the opsonic capacity of serum. Heat inactivation in this context increased the coefficient of variation significantly, which may represent the variable presence or activity of antibodies and could represent another measurable outcome.

However, heat inactivated serum also resulted in impaired mycobacterial metabolism in the restriction assay's control condition (serum + medium only; see Supplementary Figure 4). This effect, combined with reduced neutrophil phagocytosis, makes it more difficult to identify the anti-mycobacterial effect of the cells. Hence for the purposes of the restriction assay and to ensure measurement of appreciable internalisation in the phagocytosis assay, non-heat-inactivated serum is preferred in most circumstances.

In summary, we have demonstrated robust and easily used techniques to measure mycobacterial restriction and phagocytosis of mycobacteria by human neutrophils. We believe that these assays can be used to further explore the role of neutrophils in the host response to tuberculosis and the impact of potential modulators on neutrophil-mediated antimycobacterial activity.

## Figures and Tables

**Table 1:** Results from the phagocytosis assay (six donors) according to multiplicity of infection.

Multiplicity of infection (CFU: cells)	A. Neutrophils with internalised organisms only (mean % ± standard deviation)	B. Neutrophils with both internalised and external organisms (mean % ± standard deviation)	C. Neutrophils with external organisms only (mean % ± standard deviation)	D. Total with internalised organisms (=A. + B.) (mean % ± standard deviation)	E. Total with external organisms (=B. + C.) (mean % ± standard deviation)	F. Neutrophils not associated with organisms (mean % ± standard deviation)
0.03:1	5.32 ± 2.45	0.68 ± 0.24	13.05 ± 8.06	6.01 ± 2.58	13.73 ± 8.15	80.96 ± 7.15
0.15:1	20.82 ± 11.63	1.77 ± 0.65	7.17 ± 3.58	22.59 ± 12.15	8.94 ± 3.20	70.25 ± 8.85
0.3:1	31.07 ± 10.91	4.47 ± 2.33	8.51 ± 4.34	35.54 ± 11.24	12.98 ± 5.08	55.93 ± 7.50
3:1	56.42 ± 15.11	20.05 ± 15.85	3.98 ± 2.61	76.47 ± 14.62	24.03 ± 16.50	19.57 ± 13.54
