# Supplementary material for: A novel assay of antimycobacterial activity and phagocytosis by human neutrophils
Source: Tuberculosis (Edinb). 2013 Mar;93(2):167–78. doi: 10.1016/j.tube.2012.11.014 (PMC3608034; doi:10.1016/j.tube.2012.11.014)

Supplementary Figure Legends

Suplementary Figure 1. a. Time to peak luminescence after addition of substrate was measured for *M. tb-lux* incubated for one hour in either 400mcl RPMI-1640 plus 50mcl serum ('Serum') or into 400mcl neutrophil suspension (at 1 x 10^6 / ml) plus 50mcl serum ('Cells'). Column heights represent mean values from three separate donors; error bars represent SD. p = 0.001 by two-tailed t-test. b. Total measured luminescence of BCG-*lux* according to time of measurement (one experiment). Triangles = serum only samples; squares = samples containing cells. Markers represent mean values from duplicate (serum) or triplicate (cell) readings.

Supplementary Figure 2. The phagocytosis assay was performed using an MOI of 1 RLU:1 cell (0.3 CFU:1 cell); samples were incubated for 30 minutes either at 37°C or on ice (0°C). The y-axis represents the total percentage of neutrophils with internalised organisms.

Supplementary Figure 3. a. *pH sensitivity of pHrodo-labelled BCG*. BCG-lux were labelled with pHrodo™ (Invitrogen) at a concentration of 25mM according to the manufacturer's instructions, except for omission of the 100% methanol step. Approximately 100,000 CFU were resuspended in 500mcl buffer at pH 4, 7, 8 or 9.6. Samples were then acquired on a BD Fortessa flow cytometer, and pHrodo fluorescence was measured in the PE-Texas Red channel. Green line – pH9.6, orange line – pH 8, blue line – pH 7, red line – pH 4. b. *Gating strategy for pHrodo-based phagocytosis assay*. First, singlet signals are gated (forward scatter area versus height). Subsequently, dead cells are excluded using eFluor450 Viability Dye versus side scatter. Neutrophils are gated via forward and side scatter characteristics. Finally, pHrodo signal is detected in the PE-Texas Red channel versus APC (redundant channel). FSC = forward scatter, SSC = side scatter.

Supplementary Figure 4. Serum is required for mycobacterial viability. 50mcl BCG-lux was incubated into 400mcl RPMI-1640 plus 50mcl serum pre-incubated at room temperature for 30 minutes ('Room temp'), into 400mcl RPMI-1640 plus 50mcl serum pre-incubated at 56°C for thirty minutes ('56 degrees'), into 400mcl RPMI-1640 plus 50mcl serum pre-incubated at 90 degrees for 2 minutes ('90 degrees'), or into 450mcl RPMI-1640 without serum ('RPMI only'). Luminescence was measured after one hour's incubation. Markers represent the mean of duplicate results from eight separate donors (serum conditions) and three contemporaneous RPMI-only experiments.

Supplementary Figures

Supplementary Figure 1

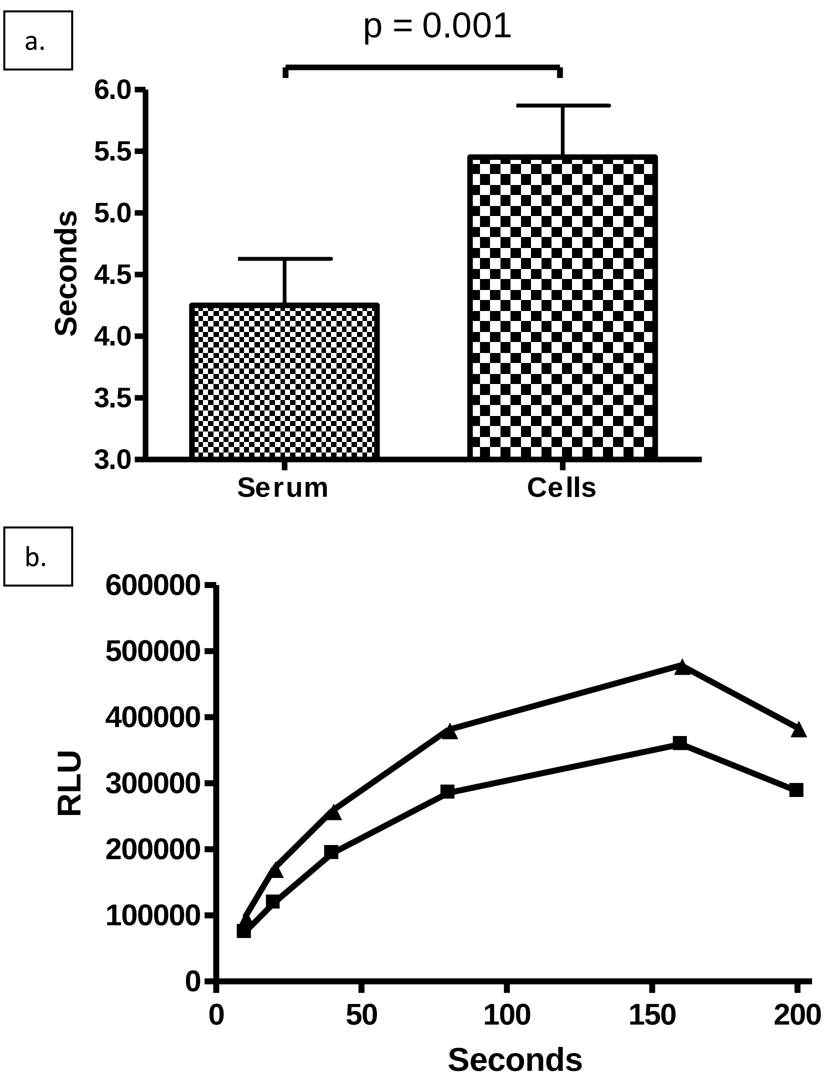

Supplementary Figure 2

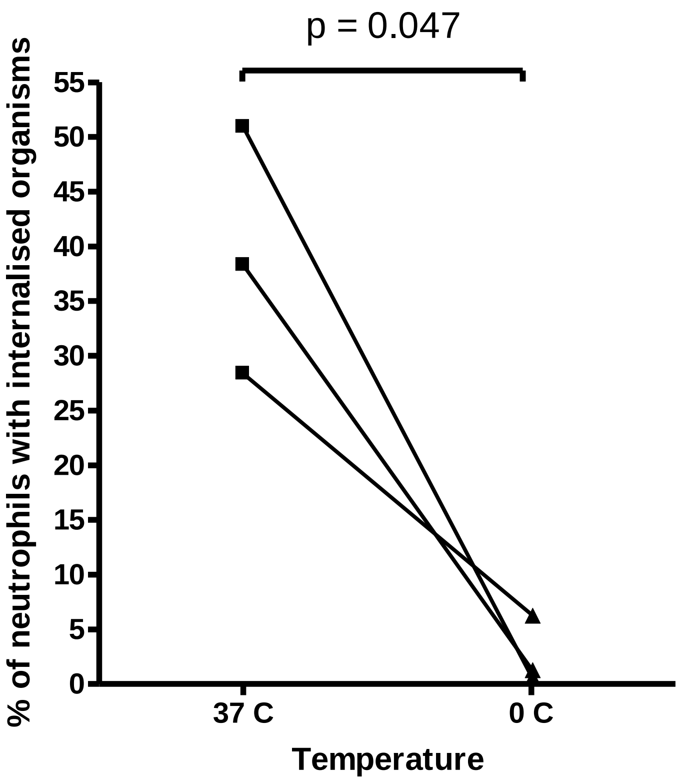

Supplementary Figure 3

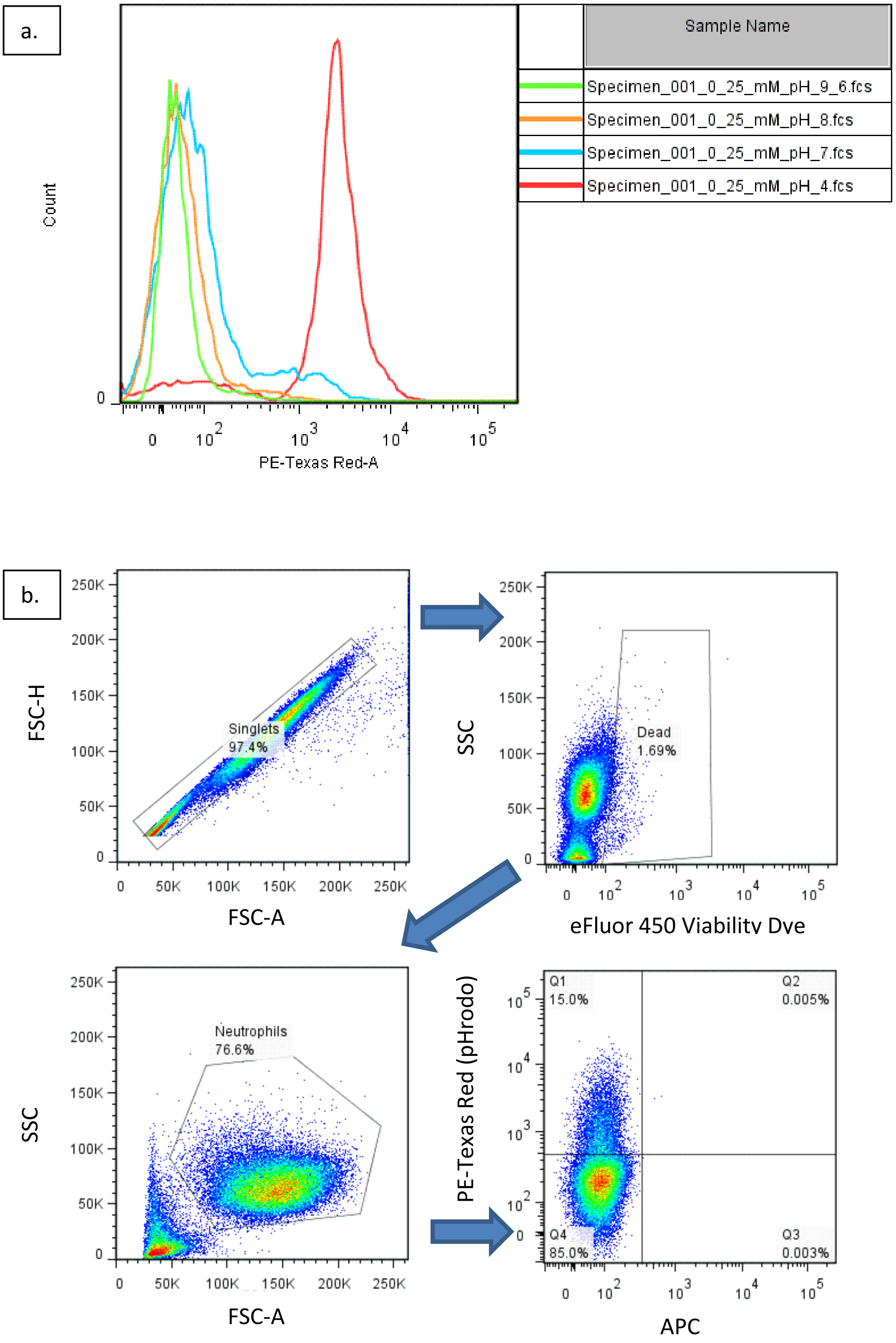

Supplementary Figure 4

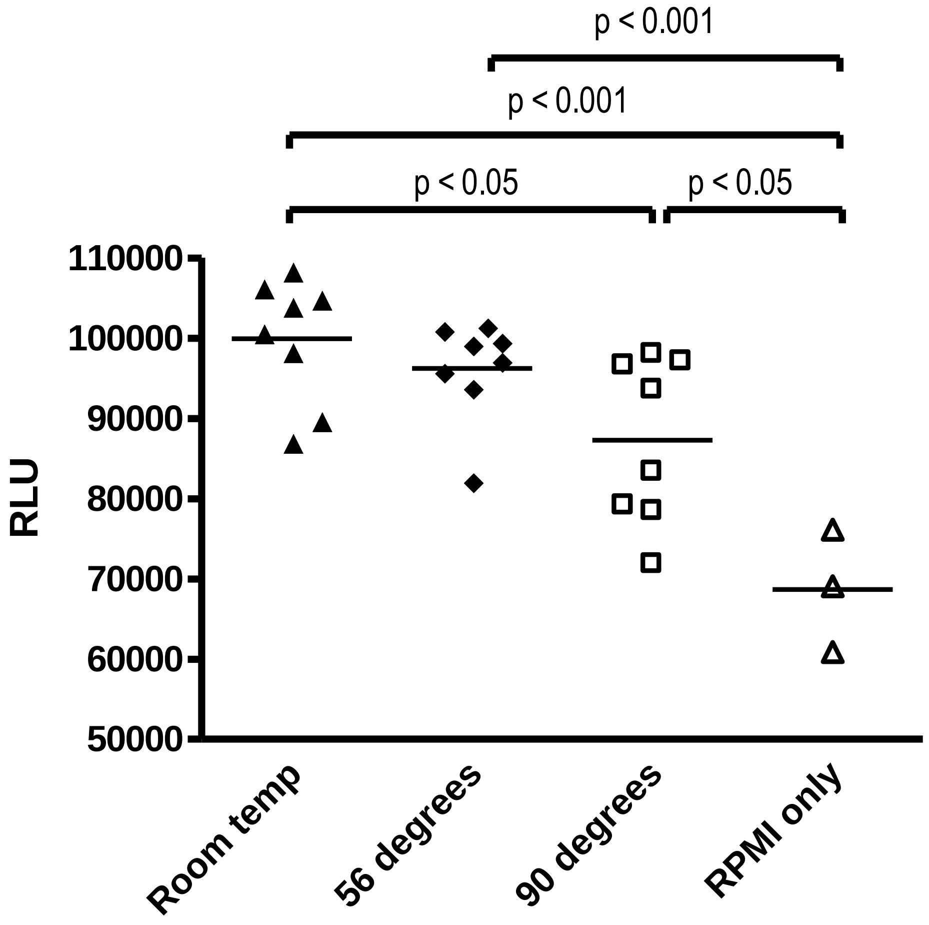

Supplement: Supplementary file 1 [file mmc1.pdf]
